# Deaths From *Plasmodium knowlesi* Malaria: Case Series and Systematic Review

**DOI:** 10.1093/cid/ciz011

**Published:** 2019-01-08

**Authors:** Giri S Rajahram, Daniel J Cooper, Timothy William, Matthew J Grigg, Nicholas M Anstey, Bridget E Barber

**Affiliations:** 1 Infectious Diseases Unit, Queen Elizabeth Hospital, Malaysia; 2 Infectious Diseases Society Kota Kinabalu Sabah-Menzies School of Health Research Clinical Research Unit, Malaysia; 3 Global and Tropical Health Division, Menzies School of Health Research and Charles Darwin University, Darwin, Northern Territory, Australia; 4 Jesselton Medical Centre, Kota Kinabalu, Sabah, Malaysia; 5 QIMR Berghofer Medical Research Institute, Brisbane, Australia

**Keywords:** knowlesi, malaria, deaths, pregnancy, sex differences

## Abstract

**Background:**

*Plasmodium knowlesi* causes severe and fatal malaria, and incidence in Southeast Asia is increasing. Factors associated with death are not clearly defined.

**Methods:**

All malaria deaths in Sabah, Malaysia, from 2015 to 2017 were identified from mandatory reporting to the Sabah Department of Health. Case notes were reviewed, and a systematic review of these and all previously reported fatal *P. knowlesi* cases was conducted. Case fatality rates (CFRs) during 2010–2017 were calculated using incidence data from the Sabah Department of Health.

**Results:**

Six malaria deaths occurred in Sabah during 2015–2017, all from *P. knowlesi*. Median age was 40 (range, 23–58) years; 4 cases (67%) were male. Three (50%) had significant cardiovascular comorbidities and 1 was pregnant. Delays in administering appropriate therapy contributed to 3 (50%) deaths. An additional 26 fatal cases were included in the systematic review. Among all 32 cases, 18 (56%) were male; median age was 56 (range, 23–84) years. Cardiovascular-metabolic disease, microscopic misdiagnosis, and delay in commencing intravenous treatment were identified in 11 of 32 (34%), 26 of 29 (90%), and 11 of 31 (36%) cases, respectively. The overall CFR during 2010–2017 was 2.5/1000: 6.0/1000 for women and 1.7/1000 for men (*P* = .01). Independent risk factors for death included female sex (odds ratio, 2.6; *P* = .04), and age ≥45 years (odds ratio, 4.7; *P* < .01).

**Conclusions:**

Earlier presentation, more rapid diagnosis, and administration of intravenous artesunate may avoid fatal outcomes, particularly in females, older adults, and patients with cardiovascular comorbidities.

## Teaser

Six deaths from *Plasmodium knowlesi* malaria in Sabah, Malaysia in 2015–2017 and 26 previously reported deaths were reviewed. Associated factors included female sex; age ≥45 years; cardiovascular-metabolic comorbidities; and delays in presentation, diagnosis, and treatment.

*Plasmodium knowlesi*, a zoonotic parasite of long-tailed and pig-tailed macaques, is an important cause of human malaria in Southeast Asia [1–3]. In some areas in this region, incidence has increased alongside the near-elimination of falciparum and vivax malaria, with *P. knowlesi* now the most common cause of malaria in Malaysia [4] and parts of western Indonesia [5–7].

Risk of severe disease from *P. knowlesi* is at least as high as from *Plasmodium falciparum* in coendemic areas [8, 9], occurring in 6%–9% of symptomatic adults [10, 11], and fatal cases have been reported [4, 9, 11–16]. Independent risk factors for severe malaria include older age and increasing parasitemia [10, 17]; however, risk factors for death are poorly defined.

To delineate factors associated with knowlesi malaria mortality, we reviewed malaria deaths in Sabah, Malaysia, from 2015 to 2017, and conducted a systematic review of all previously reported fatal cases. *Plasmodium knowlesi* case fatality rates (CFRs) in Sabah during 2010–2017 were calculated using previously reported mortality and incidence data [4, 18].

## METHODS

### Case Series: Malaria Deaths, Sabah, 2015–2017

All malaria deaths in Sabah from 2015–2017 were identified from the Sabah Department of Health. In Sabah, hospital admission and notification to the Department of Health are mandatory for all malaria cases, as is reporting of deaths. Since 2015 it has also been mandatory for a blood sample from all malaria cases to be sent to the state public health laboratory for *Plasmodium* species confirmation by polymerase chain reaction (PCR).

Case notes were retrieved from relevant health facilities and reviewed. Approval was obtained from the ethics committees of the Ministry of Health, Malaysia, and the Menzies School of Health Research, Australia.

### Systematic Review of the Literature

We searched Medline, PubMed, and Science Direct for articles published up to August 2018, containing the words “knowlesi AND death,” “knowlesi AND fatality,” or “knowlesi AND outcome,” including synonyms and Medical Subject Heading terms. Relevant references cited within these articles were also reviewed.

### Case Fatality Rates

*Plasmodium* *knowlesi* CFRs in Sabah were calculated from 2010 to 2017 using malaria incidence and fatality data from the Sabah Department of Health [4, 18]. Male and female CFRs were compared using Fisher exact test. Logistic regression was used to evaluate age and sex as independent risk factors for death.

## RESULTS

### Case Series: Malaria Deaths, Sabah, 2015–2017

Six malaria deaths were reported to the Sabah Department of Health during 2015–2017, all confirmed by PCR as *P. knowlesi* monoinfections. Baseline demographic and clinical details are shown in Tables 1 and 2, laboratory details in Table 3, and severity criteria in Table 4 (cases G1–G6). The median age was 40 (range, 23–58) years. Four (67%) cases were male; 3 (50%) were non-Malaysian citizens. Three (50%) cases had significant cardiovascular-metabolic comorbidities (severe mitral stenosis, ischemic heart disease, and morbid obesity with heart failure, respectively), and one other was pregnant. Median fever duration was 8 (range, 4–14) days. Five had severe malaria on presentation; intravenous (IV) artesunate was administered upon hospitalization in only 2 cases, with artesunate unavailable in 2 cases. Initial hospital microscopy correctly reported *P. knowlesi* in 2 (33%) cases; the others were reported as *Plasmodium malariae* (n = 2) and *P. falciparum* (n = 2). Median time from admission to death was 29 (range, 7–144) hours.

**Table 1.** Article Summary, Demographics, and Treatment

Author	Year Published (Period)	Location	Case	Sex	Age, y	Fever, d	Malaysian Citizen	Correct Diagnosis^a^	Severe Diagnosis^b^	Initial Therapy IV	Therapy^c^	Time to Death, h
Cox-Singh et al [14]	2008 (2001–2006)	Sarawak	A1	F	66	3	NR	N	N	N	CQ, SP	35
			A2	M	69	3	NR	Y	Y	NR	Q	141
			A3	M	39	3	NR	Y	Y	N	CQ, SP	5
			A4	M	40	7	NR	Y	Y	Y	CQ, SP, PQ, Q	316
Daneshvar et al [11]	2009 (2006–2008)	Sarawak	B1	F	68	NR	NR	NR	Y	Y	Q	6
			B2	F	36	NR	NR	NR	Y	Y	Q	24
Cox-Singh et al [16]	2010	Sarawak	C1	M	40	5	Y	N	N	N	None	2
William et al [9]	2011 (2007–2009)	Sabah	D1	F	76	NR	Y	NR	Y	Y	Q	96
			D2	F	65	5	Y	NR	Y	Y	Q	96
			D3	M	57	7	Y	NR	Y	Y	Art	48
			D4	F	84	5	Y	NR	Y	Y	Q	96
			D5	M	56	5	Y	NR	Y	Y	Q	<24
			D6	M	46	7	Y	NR	Y	Y	Q	24
Rajahram et al [12]	2013 (2010–2011)	Sabah	E1	M	71	7	NR	Y	N	Y	Q, PQ, CQ, D	1
			E2	M	65	2	NR	N	N	N	CQ	75
			E3	M	51	4	NR	Y	Y	Y	Art	16
			E4	M	50	7	NR	Y	Y	N	CQ	56
			E5	F	49	4	NR	Y	N	N	CQ, PQ	63
			E6^d^	F	36	7	NR	Y	N	N	SP, PQ	84
Rajahram et al^e^ [4]	2016 (2012–2014)	Sabah	F1	M	31	7	Y	N	N	Y	Art, D	216
			F2^f^	F	71	5	Y	Y	Y	Y	Art, D	12
			F3	F	61	7	Y	Y	Y	Y	Art	7
			F4	M	56	3	N	Y	Y	Y	Art, D	41
			F5	F	57	7	N	Y	N	Y	Art, PQ, CQ	NR
			F6	M	73	7	Y	Y	NR	Y	Art, D	72
			F7	F	62	6	Y	Y	N	N	A-M	23
Newly reported	2018 (2015–2017)	Sabah	G1^g^	F	32	14	Y	Y	Y	N	A-L, D^h^	10.5
			G2^i^	M	50	7	N	Y	Y	Y	Art	7
			G3	F	37	4	Y	N	N	N	None	43
			G4	M	42	5	N	Y	N	N	A-L	15
			G5	M	32	9	Y	Y	Y	Y	Art	144
			G6	M	58	9	N	Y	Y	Y^h^	Art^h^	70
Total (%)			…	M: 18/32 (56)	56^j^	6^j^	15/20 (75)	…	19/24 (79)	20/31 (65)	…	41^j^

Abbreviations: A-L, artemether-lumefantrine; A-M, artemether-mefloquine; Art, artesunate; CQ, chloroquine; D, doxycycline; F, female; IV, intravenous; M, male; N, no; NR, not reported; PQ, primaquine; Q, quinine; SP, sulfadoxine-pyrimethamine; Y, yes.

^a^Diagnosed as malaria at presentation.

^b^Diagnosed as severe malaria at presentation.

^c^Initial therapy on malaria diagnosis.

^d^ *Plasmodium knowlesi–*associated, gram-negative sepsis.

^e^One fatal case of microscopy-diagnosed “*Plasmodium malariae*” was described in this series, but has not been included in the review due to lack of polymerase chain reaction confirmation.

^f^This patient was also described in a separate case report [13].

^g^Thirty-five weeks’ gestation at presentation.

^h^IV therapy not available at presenting hospital.

^i^This patient was previously reported in a prospective observational study [10].

^j^Values are presented as the median.

**Table 3.** Laboratory Features on Presentation

Case	Hb, g/dL	WBC, × 10^3^ Cells/µL	Plt, × 10^9^ Cells/µL	Na, mmol/L	Creat, µmol/L	Urea, µmol/L	Bili, µmol/L	AST, U/L	ALT, U/L	Alb, g/L	HCO_3_, mmol/L	Lac, mmol/L	Species^a^	Parasites/µL Blood
A1	10.6	16.7	22	NR	500	61	79	122	104	24	NR	NR	Pm	204 800
A2	15.2	6.6	25	NR	NR	26	300	163	77	15	NR	NR	Pm	75 000
A3	15.4	13.4	24	NR	NR	19	NR	NR	NR	NR	NR	NR	Pm	112 000
A4	11.9	11.4	24	NR	557	25	490	87	82	28	NR	NR	Pm	++++
B1	NR	NR	NR	NR	320	NR	NR	NR	NR	NR	NR	17.4	NR	222 570
B2	NR	NR	NR	NR	NR	NR	178	NR	NR	NR	NR	NR	NR	214 000
C1	12.7	10.1	43	126	NR	34	NR	131	NR	NR	NR	NR	NR	NR
D1	14.6	6.1	42	129	173	29	660	110	145	28	20	NR	Pm	4+
D2	12.6	20.1	26	128	492	50	103	67	85	32	17	NR	Pm	4+
D3	9.4	20	35	133	819	73	397	19	230	24	10.7	NR	Pm	4+
D4	10.5	9.7	34	129	239	NR	NR	NR	NR	NR	NR	NR	Pm	2+
D5	15.4	11.9	28	128	380	NR	NR	NR	NR	NR	11	NR	Pm	4+
D6	16.8	21.6	16	134	526	23	640	210	275	26	12	NR	Pm	4+
E1	14.9	10.8	88	NR	1451	82	NR	42	NR	NR	NR	NR	Pf	3+
E2	14.6	4.8	58	NR	NR	6	NR	NR	NR	NR	NR	NR	Pm	3+
E3	9.4	6.8	8	NR	578	44	146	53	28	23	14	6.4	Pm	4+
E4	10.9	7.81	3	NR	330	39	74	NR	49	19	16.2	6.8	Pv	4+
E5	12.6	12.1	32	NR	283	25	25	39	20	28	14	NR	Pm	4+
E6	11.1	7.8	53	NR	NR	11	NR	NR	NR	NR	6.5	NR	Pm	1+
F1	12.9	6.9	25	NR	915	38	254	67	NR	28	11.5	3.6	Pf	92 500
F2	14.1	13.3	53	NR	662	36	108	322	145	24	4.2	NR	Pm	71 939
F3	12.6	20.5	8	NR	453	34	315	732	258	20	10.3	NR	Pm	320 000
F4	13.8	12.1	73	NR	171	12	43	373	143	24	11.6	NR	Pk	6471
F5	11.1	5.3	26	NR	143	12	46	NR	NR	27	14.3	NR	Pv	9866
F6	11	8.2	67	NR	132	14	234	61	36	20	16	NR	Pm	4+
F7	12	11.2	60	NR	124	6	NR	NR	NR	NR	NR	NR	Pm	22 666
G1	15.8	19.5	49	123	131	11	NR	NR	NR	NR	NR	NR	Pk	22 400
G2	9.9	8.5	63	121	609	50	NR	NR	NR	NR	NR	NR	Pk	71 939
G3	13.2	12	159	118	326	15	32	37	24	31	12.7	NR	Pf	3+
G4	13.1	7.6	44	123	481	33	36	122	57	18	12.8	NR	Pm	1+
G5	14.3	6.4	76	128	120	9	38	21	14	NR	22.3	NR	Pm	4+
G6	8.3	18	104	128	230	27	84	54	31	26	15	1.96	Pf	++++
Median	12.7	11	38.5	128	355	26.5	108	77	79.5	24	12.8	6.4	…	…

Blood culture results were available in 14 cases: 13 were negative at 3 days of incubation, and 1 (E6) grew *Enterobacter cloacae*. ++++ indicates >4,000 parasites/µL.

Abbreviations: Alb, albumin; ALT, serum alanine aminotransferase; AST, serum aspartate aminotransferase; Bili, serum bilirubin; creat, serum creatinine, Hb, plasma hemoglobin; HCO_3_, serum bicarbonate; Lac, serum lactate; Na, serum sodium; NR, not reported; Pf, *Plasmodium falciparum*; Pm, *Plasmodium malariae*; Pk, *Plasmodium knowlesi*; Plt, platelet count; Pv, *Plasmodium vivax*; Urea, serum urea; WBC, white blood cell count.

^a^ *Plasmodium* species by microscopy.

**Table 4.** Severity Criteria on Presentation

Case	Anemia	AKI	Hypoglycemia	Acidosis	Hyperparasitemia	Jaundice	Coma^a^	Shock	Bleeding	Respiratory Distress	Total
A1	N	Y	NR	NR	Y	Y	N	N	N	N	3
A2	N	NR	NR	NR	N	Y	N	N	N	Y	2
A3	N	NR	NR	NR	Y	Y	N	N	N	N	2
A4	N	Y	NR	NR	Y	Y	N	N	N	N	3
B1	N	Y	Y	Y	Y	Y	NR	Y	NR	Y	7
B2	N	N	N	N	Y	Y	NR	N	NR	Y	3
C1	N	NR	NR	NR	Y	NR	N	Y	N	Y	3
D1	N	Y	Y	Y	NR	Y	N	Y	N	Y	6
D2	N	Y	N	Y	NR	Y	N	Y	N	Y	5
D3	N	Y	Y	Y	NR	Y	N	Y	N	Y	6
D4	N	Y	N	N	NR	N	N	N	N	Y	2
D5	N	N	N	N	NR	N	N	Y	N	Y	2
D6	N	Y	N	N	NR	Y	N	N	N	Y	3
E1	N	Y	NR	NR	NR	NR	N	Y	NR	Y	3
E2	N	NR	NR	NR	NR	NR	N	N	N	N	0
E3	N	Y	NR	Y	NR	Y	N	Y	N	Y	5
E4	N	Y	NR	Y	NR	Y	N	N	N	Y	4
E5	N	Y	NR	Y	NR	N	N	N	N	Y	3
E6	N	NR	NR	Y	N	NR	N	N	N	N	1
F1	N	Y	NR	Y	N	Y	N	N	N	N	3
F2	N	Y	Y	Y	Y	Y	N	N	N	N	5
F3	N	Y	NR	Y	Y	Y	N	N	N	Y	5
F4	N	N	NR	Y	N	N	Y^b^	N	N	Y	3
F5	N	N	NR	NR	N	Y	N	N	N	N	1
F6	N	N	NR	N	NR	Y	N	Y	N	N	2
F7	N	N	NR	NR	Y	NR	N	N	N	N	1
G1	N	N	NR	NR	N	NR	N	Y	N	Y	3
G2	N	Y	NR	NR	Y	NR	N	N	N	N	2
G3	N	Y	NR	Y	NR	N	N	N	N	N	2
G4	N	Y	NR	Y	NR	N	N	Y	N	Y	4
G5	N	N	Hyper	N	N	N	N	N	N	N	0
G6	N	Y	Hyper	Y	Y	Y	N	Y	N	Y	6
Frequency, No. (%)	0/32 (0)	19/27 (70)	4/11 (36)	15/21 (71)	11/18 (61)	18/25 (72)	1/30 (3)	12/32 (38)	0 (0)	19/32 (59)	

Definitions of severe *Plasmodium knowlesi* malaria criteria: severe anemia, hemoglobin <7.0 g/dL (adults) or <5.0 g/dL (children); AKI, creatinine >265 µmol/L; hypoglycemia, blood glucose <2.2 mmol/L; metabolic acidosis, bicarbonate <15 mmol/L or lactate >5 mmol/L; hyperparasitemia, parasite count >100 000 parasites/µL (or >2% infected red blood cells); jaundice, bilirubin >50 µmol/L, with parasitemia >20 000/µL and/or creatinine >132 µmol/L; coma, Glasgow Coma Scale (GCS) <11; shock, systolic blood pressure <80 mm Hg with cool peripheries or impaired capillary refill; significant abnormal bleeding; respiratory distress, oxygen saturation <92% with respiratory rate >30 breaths/minute.

Abbreviations: AKI, acute kidney injury; Hyper, hyperglycemia; N, no; NR, not reported; Y, yes.

^a^Not reported to date in knowlesi malaria.

^b^Alternative causes for reduced GCS not excluded.

#### Case 1 (G1)

A 32-year-old pregnant woman at 35 weeks’ gestation (G6P5) presented with reduced fetal movement, 14 days of fever, and 2 days of intermittent abdominal pain and dyspnea. She was hypotensive, tachycardic, hypoxic, and tachypneic. She had hyponatremia, thrombocytopenia, and acute kidney injury (AKI). She was intubated, ventilated, and given IV ceftriaxone, sodium bicarbonate, and ionotropic support. A chest radiograph showed diffuse interstitial infiltrates consistent with acute respiratory distress syndrome (ARDS). A blood film taken 4 hours after admission was reported as *P. knowlesi*, with 22 400 parasites/µL. Oral artemether-lumefantrine and doxycycline were given, and tertiary hospital transfer was arranged for IV artesunate; however, the patient had a cardiac arrest while awaiting transfer. Admission blood cultures were negative.

#### Case 2 (G2)

A 50-year-old male Indonesian palm-oil plantation worker presented to a district hospital with 1 week of fever, rigors, headache, epigastric pain, and diarrhea. Physical examination revealed hepatomegaly (3 cm), but was otherwise unremarkable. He had severe AKI, hyponatremia, and thrombocytopenia. A blood film was reported as *P. knowlesi* with 71 939 parasites/µL. Chest radiography showed generalized interstitial infiltrates. He was diagnosed with severe malaria and commenced on IV artesunate, and a tertiary hospital transfer was arranged; however, he arrested en route. This case has been previously documented [10].

#### Case 3 (G3)

A 37-year-old woman with a history of gestational hypertension presented to a district hospital with 4 days of fever, rigors, epigastric pain, dizziness, and vomiting. She was hypotensive, tachycardic, and had epigastric tenderness. The initial diagnosis was dyspepsia, and she received ranitidine, prochlorperazine, and fluid resuscitation. AKI was noted 2 hours after admission, and IV ceftriaxone was initiated for presumed sepsis. Twelve hours after hospitalization, she became tachypneic, with reduced consciousness (Glasgow Coma Scale reported as 9/15), and severe metabolic acidosis (arterial pH, 7.02; bicarbonate, 2.5 mEq/L). She underwent intubation, ventilation, and hemodialysis and received IV sodium bicarbonate and imipenem. The patient deteriorated further on day 2, with transaminitis (alanine aminotransferase [ALT] level, 426 U/L; aspartate aminotransferase [AST] level, 1481 U/L), metabolic acidosis, and coagulopathy (international normalized ratio [INR], 1.9; prothrombin time, 52 seconds) requiring fresh frozen plasma. The patient arrested 43 hours after admission. Blood films taken prior to death were reported as *P. malariae* with 2285 parasites/µL. It was later noted that an admission blood film had been reported as “*P. falciparum* 3+.” No antimalarial treatment had been given. Blood cultures were negative.

#### Case 4 (G4)

A 32-year-old Filipino man presented with 5 days of fever, cough, and coryzal symptoms. He was tachycardic, but physical examination was otherwise unremarkable. He was given fluids and acetaminophen (paracetamol). A blood smear was reported as “*P. malariae* 1+” with AKI, hyponatremia, and thrombocytopenia also noted. Oral artemether-lumefantrine was given. Twenty-two hours after admission, the patient had hemoptysis with tachypnea and hypoxia. A chest radiograph showed generalized interstitial infiltrates. A diagnosis of severe *P. malariae* with ARDS was made and IV artesunate was initiated. After further hemoptysis, a bedside ultrasound showed pleural and pericardial effusions and noninvasive ventilation was initiated. At 48 hours, massive hemoptysis occurred. He required intensive care unit admission, intubation, ventilation, inotropic support, hemodialysis for metabolic acidosis and uremia, and red cell and fresh frozen plasma transfusions. Echocardiography (ECG) showed severe mitral stenosis. Admission blood cultures were negative. He died on day 4, with cause of death recorded as severe malaria with pulmonary hemorrhage and underlying mitral stenosis.

#### Case 5 (G5)

A 42-year-old fisherman with morbid obesity, obstructive sleep apnea, and congestive cardiac failure was referred to a district hospital from a peripheral clinic with 9 days of fever, and 3 days of dyspnea, epigastric pain, and vomiting. The clinic blood film was reported as “complicated malaria with hyperparasitemia”; *Plasmodium* species was not specified. On admission he was tachycardic, hypotensive, and hypoxic. ECG showed atrial fibrillation at 160 beats per minute. He received IV artesunate, fluid resuscitation, and high-flow oxygen. He was hyponatremic, with AKI, hyperglycemia, and a compensated metabolic acidosis. A blood film was reported as “*P. malariae* 4+.” He required intubation, hemodialysis, IV insulin, digoxin, and inotropic support. Seven hours postadmission, ECG showed cardiac ischemia, and at 15 hours the patient arrested. Admission blood cultures were negative. Cause of death was recorded as (1) malaria with hyperparasitemia, and (2) decompensated cardiac failure, with likely underlying cardiomyopathy.

#### Case 6 (G6)

A 58-year-old Caucasian expatriate man with a history of ischemic heart disease, left bundle-branch block, hypertension, and paroxysmal atrial fibrillation presented to a private hospital with 9 days of fever, palpitations, and lethargy. He was hypotensive, hypoxic, and tachypneic. A blood smear was reported as “heavy infection, likely *P. falciparum*.” He was anemic, hyperbilirubinemic, and hyponatremic, with AKI and metabolic acidosis. He was commenced on ionotropic support and transferred to a tertiary public hospital for IV artesunate. There he also received ceftriaxone; hydrocortisone; insulin infusion for hyperglycemia; cardioversion for ventricular tachycardia; and, later, intubation, ventilation, and continuous venovenous hemofiltration. A blood film on day 1 was reported as *P. knowlesi* with 246 100 parasites/µL. On day 2, he had worsening metabolic acidosis (lactate, 11.7 mmol/L; pH, 7.13; bicarbonate, 10.4 mEq/L), coagulopathy (INR, 4.53), and transaminitis (AST, >4200 U/L; ALT, 1820 U/L), with a bilirubin of 247 μmol/L, and was anuric. He died on day 3, with cause of death recorded as severe knowlesi malaria with multiorgan failure.

### Systematic Review of *P. knowlesi* Fatalities

**Figure 1.**
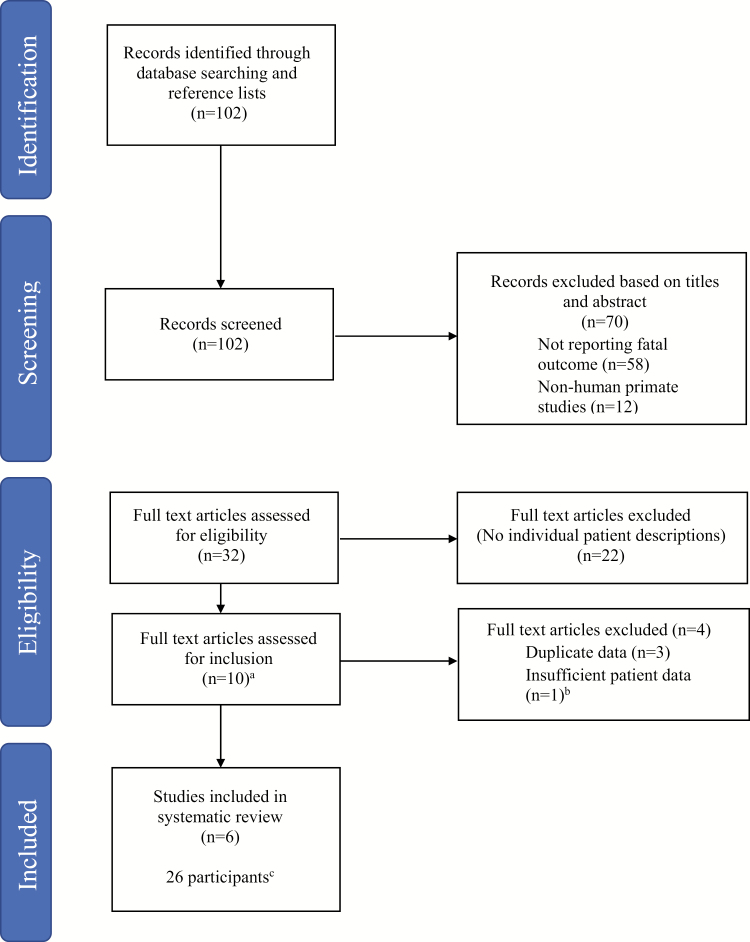
Flow diagram of systematic review. ^a^Four retrospective case series or studies [4, 9, 12, 14], 2 prospective observational studies [10, 11], 1 case-control study [15], 2 case reports [13, 16], and 1 review of autopsies [35]. ^b^Four reported deaths were excluded from further review as sufficient individual patient data were not available [15]. ^c^Nineteen deaths occurred in Sabah and 7 in Sarawak, Malaysia, all during 2001–2014.

Ten original research articles reporting *P. knowlesi* fatalities were identified (Figure 1). Thirty PCR-confirmed *P. knowlesi* deaths were reported: 19 from Sabah and 11 from Sarawak, Malaysia, all occurring during 2001–2014. Four of the Sarawak deaths were excluded from further review due to insufficient individual patient data [15].

Demographic, clinical, and laboratory details for each case are summarized in Tables 1–3, with severity criteria shown in Table 4. Combining these 26 previously reported cases with the 2015–2017 series, median age was 56 (range, 23–84) years, and 18 (56%) were male. Female fatal cases were older than male fatal cases (median age, 62 vs 51 years, respectively), although this was not statistically significant. Median duration of fever was 6 (interquartile range, 4–7) days, with no difference between women and men. At presentation, 30 of 32 (94%) patients met the World Health Organization (WHO) criteria for severe malaria, although only 19 of these 30 (63%) were recognized as having severe malaria. Of those meeting severity criteria, the median number of criteria was 3 (range, 1–7), with the most frequent criteria being respiratory distress (n = 19/32 [59%]), jaundice (n = 18/25 [72%]), and severe AKI (n = 19/27 [70%]). Abdominal pain was reported in 17 of 26 (65%) cases. Decreased conscious state was reported on presentation in 1 case; however, alternative causes were not excluded [4]. Cardiovascular-metabolic comorbidities were a notable factor of previously reported cases as well as in the current series. Overall, hypertension was reported in 8 of 32 (25%) cases and stress hyperglycemia or diabetes mellitus in 12%.

**Table 2.** Clinical Features on Presentation

Case	BP, mm Hg	HR, Beats/Min	O_2_, %	RR, Breaths/Min	Temperature, °C	Abdominal Pain^a^	Comorbidities or Pregnancy
A1	120/90	88	NR	NR	36.8	Y	…
A2	124/66	132	NR	NR	38.0	Y	…
A3	81/51	84	NR	NR	36.0	Y	…
A4	132/67	84	NR	NR	36.0	Y	…
B1	80 sys	NR	NR	NR	NR	NR	…
B2	NR	NR	NR	NR	NR	NR	HTN
C1	U	NR	NR	NR	NR	Y	…
D1	126/70	130	85	NR	36.8	NR	IHD
D2	136/57	101	56	NR	38.0	N	NR
D3	100/50	110	90	NR	36.7	Y	NR
D4	120/69	94	NR	NR	38.5	N	NR
D5	90/50	130	89	NR	37.5	NR	NR
D6	80/50	120	90	NR	37.9	Y	NR
E1	75/49	127	75	NR	39.4	NR	HTN, COPD
E2	117/73	58	92	NR	36.8	N	HTN, T2D
E3	70/40	110	90	NR	37.6	Y	…
E4	83/51	89	60	NR	37.5	N	…
E5	112/51	113	88	NR	36.7	NR	…
E6	131/87	110	85	NR	37.8	Y	…
F1	129/72	108	97	24	37.4	Y	…
F2	127/87	108	96	38	37.0	Y	HTN
F3	112/79	124	88	26	36.8	Y	HTN
F4	85/60	150	81	25	36.2	N	…
F5	193/96	129	98	22	39.0	N	HTN, T2D
F6	80/49	72	100	20	37.7	N	TB
F7	116/71	111	96	22	39.9	N	HTN
G1	69/50	138	71	46	37.0	Y	Pregnancy
G2	106/64	80	96	18	36.8	Y	…
G3	96/36	104	100	16	36.6	Y	…
G4	U	126	98^b^	20	36.3	N	Severe MR, TR
G5	105/64	129	98	26	37.6	Y	Morbid obesity, CCF, OSA
G6	78/45	86	88	27	39.7	Y	IHD, HTN, PAF
Median	…	110	90	24	37.4	…	…

Abbreviations: BP, blood pressure; CCF, congestive cardiac failure; COPD, chronic obstructive pulmonary disease; HR, heart rate; HTN, hypertension; IHD, ischemic heart disease; MR, mitral valve regurgitation; N, no; NR, not reported; O_2_, oxygen saturation; OSA, obstructive sleep apnea; PAF, paroxysmal atrial fibrillation; RR, respiratory rate; Sys, systolic; T2D, type 2 diabetes mellitus; TB, tuberculosis; TR, tricuspid valve regurgitation; U, unrecordable; Y, yes.

^a^Previously demonstrated as a risk factor for severe malaria.

^b^Fifteen liters/minute high-flow oxygen.

Among all fatal cases, species diagnosis on admission microscopy was incorrect in 26 of 29 (90%), including 20 (69%) diagnosed as *P. malariae*, 4 (14%) as *P. falciparum*, and 2 (7%) as *Plasmodium vivax*. Thrombocytopenia was universal on presentation, as was elevated creatinine (Table 3). All patients with an available serum sodium result were hyponatremic. No patient had severe anemia at presentation. Intravenous antimalarial treatment was administered on presentation in 17 of 21 (81%) cases with severe malaria on presentation. Median time to death was 41 (range, 1–316) hours.

### Case Fatality Rates

The *P. knowlesi* CFR in Sabah was 1.70/1000 (95% confidence interval [CI], 1.66–1.75; 6/3524) during 2015–2017. For women this was 3.6/1000 (95% CI, 3.4–3.8; 2/558), compared to 1.4/1000 (95% CI 1.3–1.4; 4/2966) for men (*P* = .24). Incorporating previously reported data from 2010 to 2014 (6 deaths among 783 *P. knowlesi* cases in women; 7 deaths among 3443 cases in men) [4], the overall CFR during 2010–2017 was 2.45/1000 cases (95% CI, 2.42–2.49; 19/7750): 6.0/1000 (95% CI 5.9–6.1; 8/1341) for women and 1.7/1000 (95% CI, 1.7–1.8; 11/6409) for men (*P* = .01). The higher CFR in women remained significant after adjusting for age (odds ratio [OR], 2.6 [95% CI, 1.0–6.7]; *P* = .043). The CFR for patients aged ≥45 years was 5.8/1000 (95% CI, 5.7–5.9; 13/2228) compared to 1.1/1000 (95% CI, 1.0–1.1; 6/5514) for those aged <45 years (*P* < .001). Age ≥45 years remained a significant risk factor for death after adjusting for sex (OR, 4.7 [95% CI, 1.8–12.5]; *P* = .002).

## DISCUSSION

We describe 6 malaria deaths in Sabah during 2015–2017, and review 26 previously reported *P. knowlesi* deaths. *Plasmodium knowlesi* accounted for all recent malaria deaths in Sabah, in contrast to previous reports during 2010–2014 where 13 of 29 (44%) deaths were attributed to *P. knowlesi* [4, 12]. This increase in proportion of fatal cases attributed to *P. knowlesi* is consistent with the decline of falciparum and vivax malaria in Sabah, and the ongoing increase in cases of *P. knowlesi* [4, 18]. The fact that the total number of *P. knowlesi* deaths per year has remained relatively stable despite this increasing incidence is consistent with previously reported declining CFRs in Sabah [4], likely reflecting ongoing increases in awareness and improved management of severe knowlesi malaria.

It is notable that all reported fatal cases were adults. This is consistent with the absence of severe knowlesi malaria in children [10, 19], and is in marked contrast to the predominance of severe and fatal malaria from *P. falciparum* and *P. vivax* in the pediatric age group [20]. Older age is a known risk factor for severe knowlesi malaria [8, 10, 17], and in this study age ≥45 years was associated with a 5-fold increase in risk of death. It was also notable that nearly half of all reported fatal cases were women. This contrasts with the predominance of men in studies of nonfatal knowlesi malaria, with men consistently accounting for 75%–80% of cases [21, 22]. We previously hypothesized that the higher CFR in women with knowlesi malaria was due in part to their older median age [4]. However, here we now demonstrate that women are more than twice as likely to die from knowlesi malaria even after adjusting for age. There was no difference in parasitemias between male and female fatal cases, nor any difference in fever duration, suggesting that the higher mortality was not due to delays in health-seeking behavior. A higher risk of death in females has also been noted in other severe infections [23–25]. Further studies are required to investigate the pathogenic or other factors that may account for this increased risk [24].

Respiratory distress and AKI were the most common severity criteria on presentation in fatal cases. Abdominal pain, previously reported as a risk factor for severe knowlesi malaria [10], was present in 53% of all cases. Potential explanations include gut ischemia from microvascular accumulation of parasitized red cells and/or gastric ulceration [14]. Thrombocytopenia was universal among fatal cases. This may relate to the higher proportion of platelet binding to infected red cells found in *P. knowlesi* compared with other species [26]. Hyponatremia, a known metabolic abnormality in severe falciparum and knowlesi malaria [8, 10, 27], was present in all fatal cases. Hypoglycemia occurred in 15% of previously reported fatal cases; however, in this series, 2 cases of hyperglycemia associated with death also occurred. This may relate to the older age of knowlesi-infected patients with severe malaria [8, 17], and rising prevalence of metabolic syndrome with age.

Comorbidities are a notable feature in fatal knowlesi malaria. Half of the newly described cases had underlying cardiovascular-metabolic disease (including severe mitral stenosis, heart failure, and morbid obesity), and hypertension and hyperglycemia or diabetes mellitus were also common in the previously reported cases. Cardiovascular disease and metabolic syndrome are known to be associated with increased systemic inflammation, endothelial activation, and microvascular dysfunction [28], all features of severe knowlesi malaria [17]. It is thus plausible that cardiovascular-metabolic disease would increase the risk of severe knowlesi malaria, as has been reported in falciparum malaria [29].

This series includes the first report of fatal knowlesi malaria in pregnancy, occurring at 35 weeks’ gestation. *Plasmodium knowlesi* is relatively rare in pregnancy, with only 5 cases reported previously [9, 30]. Pregnancy is known to increase risk of severe maternal disease in falciparum malaria [20]. Of the 5 previously described cases of *P. knowlesi* in pregnancy, 1 had severe malaria with intrauterine death, 2 had moderate anemia, and 1 delivered a preterm low-birth-weight infant [9, 30]. With the current fatal case, 2 of 6 (33%) reported cases of *P. knowlesi* in pregnancy have resulted in severe maternal malaria and fetal death. Prospective studies are required to further evaluate the true risks and consequences of knowlesi malaria in pregnancy.

Several health-system issues contributed to deaths in this newly reported series. In 3 cases, the diagnosis of severe malaria was delayed despite the presence of compatible clinical features, and in another 2, IV artesunate was not available. Half of the cases were non-Malaysian citizens, which, together with the long duration of fever in this series (median, 8 days), suggests possible barriers to accessing healthcare. These findings highlight the need to evaluate strategies to improve healthcare access in endemic areas, in addition to ensuring availability of appropriate antimalarials at all facilities. In the combined analysis, 90% of fatal cases had an alternative *Plasmodium* species diagnosed on admission microscopy. This is consistent with the inability of routine microscopy to reliably distinguish *P. knowlesi* from other species [31], which, combined with the poor sensitivity and specificity of available rapid diagnostic tests [32, 33], highlights the importance of a unified treatment strategy of IV artesunate for severe malaria from any species [20, 34].

A limitation of this study was the retrospective nature of the case series, resulting in either incomplete or unvalidated data. In particular, parasite counts were surprisingly low in some cases (G4 and G3), and it is possible that blood films may have been misreported. Second, the Department of Health data used to calculate CFRs did not include parasite counts, and hence the contribution of parasitemia to age and sex as risk factors for death could not be evaluated. *Plasmodium knowlesi* parasitemia is known to increase with age; however, age has been shown to be an independent risk factor for severe knowlesi malaria [10, 17], and hence would be expected to be an independent risk factor for death. In the largest prospective studies conducted to date [10, 17], an independent association between parasitemia and sex was not found; thus, a difference in parasitemia is unlikely to account for the increased CFR in women. Finally, it is possible that not all cases of *P. knowlesi* are notified, so the CFRs reported in this manuscript may overestimate the true CFRs.

In conclusion, this review highlights the potential for poor outcomes from knowlesi malaria, and identifies female sex, age ≥45 years, and comorbidities as important associated factors. With the near-elimination of falciparum and vivax malaria in Sabah, Malaysia, health systems must maintain efforts to promptly diagnose and treat patients with knowlesi malaria to avoid fatal outcomes, particularly in these at-risk groups. This will require ongoing surveillance and efforts to increase awareness among communities and health providers, as well as improving access to healthcare and ensuring availability of appropriate antimalarials at all health facilities.

## Notes

***Acknowledgments.*** The authors acknowledge the help of the medical records departments at the relevant facilitates for facilitating access to patient case notes; the Sabah Department of Health for providing malaria notification data; and the Director-General, Ministry of Health, Malaysia, for permission to publish the data. The authors thank Joseph Benedict, Clarice James, and Danshy Alaza at the Infectious Disease Society, Kota Kinabalu, Sabah, for assistance with data collection, and Tsin Wen Yeo for providing additional unpublished data on cases D1–D6.

***Financial support.*** This work was supported by the Australian National Health and Medical Research Council (fellowships to N. M. A., B. E. B., and M. J. G.), and Charles Darwin University Prestigious International Research Tuition Scholarship and University Postgraduate Research Scholarship to D. J. C.

***Potential conflicts of interest.*** All authors: No reported conflicts. All authors have submitted the ICMJE Form for Disclosure of Potential Conflicts of Interest. Conflicts that the editors consider relevant to the content of the manuscript have been disclosed.
